# Carbapenem resistant *Pseudomonas aeruginosa* and *Acinetobacter baumannii* at Mulago Hospital in Kampala, Uganda (2007–2009)

**DOI:** 10.1186/s40064-016-2986-7

**Published:** 2016-08-09

**Authors:** David P. Kateete, Ritah Nakanjako, Juliet Namugenyi, Joseph Erume, Moses L. Joloba, Christine F. Najjuka

**Affiliations:** 1 Department of Immunology and Molecular Biology, College of Health Sciences, Makerere University, Kampala, Uganda; 2 Department of Medical Microbiology, College of Health Sciences, Makerere University, Kampala, Uganda; 3 College of Veterinary Medicine, Animal Resources and Biosecurity, Makerere University, Kampala, Uganda; 4 Makerere University-Johns Hopkins University (MU-JHU) Core Lab at the Infectious Diseases Institute (IDI), College of Health Sciences, Makerere University, Kampala, Uganda

**Keywords:** Carbapenemase genes, Metallo-beta-lactamases, OXA-carbapenemases, Class 1 integrons, Hospital environment

## Abstract

**Background:**

Multidrug resistant *Pseudomonas aeruginosa* and *Acinetobacter baumannii* are common causes of health care associated infections worldwide. Carbapenems are effective against infections caused by multidrug resistant Gram-negative bacteria including *Pseudomonas* and *Acinetobacter* species. However, their use is threatened by the emergence of carbapenemase-producing strains. The aim of this study was to determine the prevalence of carbapenem-resistant *P. aeruginosa* and *A. baumannii* at Mulago Hospital in Kampala Uganda, and to establish whether the hospital environment harbors carbapenem-resistant Gram-negative rods.

**Results:**

Between February 2007 and September 2009, a total of 869 clinical specimens were processed for culture and sensitivity testing yielding 42 (5 %) *P. aeruginosa* and 29 (3 %) *A. baumannii* isolates, of which 24 % (10/42) *P. aeruginosa* and 31 % (9/29) *A. baumannii* were carbapenem-resistant. Additionally, 80 samples from the hospital environment were randomly collected and similarly processed yielding 58 % (46/80) *P. aeruginosa* and 14 % (11/80) *A. baumannii*, of which 33 % (15/46) *P. aeruginosa* and 55 % (6/11) *A. baumannii* were carbapenem-resistant. The total number of isolates studied was 128. Carbapenemase genes detected were *bla*_IMP_-like (36 %, 9/25), *bla*_VIM_-like (32 %, 8/25), *bla*_SPM_-like (16 %, 4/25); *bla*_NDM-1_-like (4 %, 1/25) in carbapenem-resistant *P. aeruginosa*, and *bla*_OXA-23_-like (60 %, 9/15), *bla*_OXA-24_-like (7 %, 1/15), *bla*_OXA-58_-like (13 %, 2/15), and *bla*_VIM_-like (13 %, 2/15) in carbapenem-resistant *A. baumannii*. Furthermore, class 1 integrons were detected in 38 % (48/128) of *P. aeruginosa* and *Acinetobacter*, 37 % (26/71) of which were in clinical isolates and 39 % (22/57) in environment isolates. Gene cassettes were found in 25 % (12/48) of integron-positive isolates. These were aminoglycoside adenylyltransferase *ant(4′)*-*IIb* (3 isolates); trimethoprim-resistant dihydrofolate reductase *dfrA* (2 isolates); adenyltransferase *aadAB* (3 isolates); *QacE* delta1 multidrug exporter (2 isolates); quinolone resistance pentapeptide repeat protein *qnr* (1 isolate); and metallo-β-lactamase genes *bla*_VIM-4_-like, *bla*_IMP-19_-like, and *bla*_IMP-26_-like (1 isolate each). Gene cassettes were missing in 75 % (36/48) of the integron-positive isolates.

**Conclusions:**

The prevalence of carbapenem-resistant *P. aeruginosa* and *Acinetobacter* among hospitalized patients at Mulago Hospital is low compared to rates from South-East Asia. However, it is high among isolates and in the environment, which is of concern given that the hospital environment is a potential source of infection for hospitalized patients and health care workers.

**Electronic supplementary material:**

The online version of this article (doi:10.1186/s40064-016-2986-7) contains supplementary material, which is available to authorized users.

## Background

Multidrug resistant *Pseudomonas aeruginosa* and *Acinetobacter baumannii* are common causes of nosocomial infections worldwide (Falagas et al. 2005; Giamarellos-Bourboulis et al. 2003; Gootz and Marra 2008; Tam et al. 2010; Turton et al. 2005). Carbapenems are the most effective drugs against infections caused by multidrug resistant Gram-negative bacteria including *Pseudomonas* and *Acinetobacter* species (Papp-Wallace et al. 2011; Manenzhe et al. 2015). However, their use in the management of these infections is threatened by the emergence of carbapenemase producing strains. Carbapenemases are β-lactamase enzymes with capacity to hydrolyze carbapenems, penicillins, and cephalosporins; they were first described in the 1990s and have continued to be reported every year with increasing rates (Papp-Wallace et al. 2011; Tenover 2006; Queenan and Bush 2007).

Carbapenemases are assigned to three of four classes of β-lactamases; Ambler classes A, B, and D that are differentiated based on the hydrolytic mechanisms at their active sites (Manenzhe et al. 2015; Queenan and Bush 2007). Class A and D carbapenemases are referred to as Serine carbapenemases because they have Serine (amino acid) at the active site (Serine-dependent), whereas class B carbapenemases have zinc (zinc-dependent) and are referred to as metallo-β-lactamases. Ambler class A carbapenemases can be plasmid encoded or chromosomal and are inhibited by clavulanic acid, a β-lactamase inhibitor; SME, IMI, NMC, GES, and KPC families are the most frequently identified class A carbapenemases mostly in *Klebsiella pneumoniae*. **Class B metallo-β-lactamases** are plasmid-encoded (in some cases chromosomal) and the most common enzymes include the VIM, IMP, SPM, GIM, SIM and NDM families. Metallo-β-lactamases have been detected primarily in *P. aeruginosa* but they are also increasingly being detected in *Acinetobacter* species (Tsakris et al. 2006) and the Enterobacteriaceae (Turton et al. 2005, 2006; Okoche et al. 2015); NDM producing Enterobacteriaceae are currently a major concern due to their rapid spread worldwide (Manenzhe et al. 2015). Furthermore, class B enzymes are able to hydrolyze β-lactams except aztreonam (a monobactam) and their hydrolytic activity is inhibited by EDTA (ethylenediammine tetra acetic acid), but not clavulanic acid. On the other hand, **class D enzymes**, also referred to as the OXA-type carbapenemases, are subdivided into five families; OXA-23, OXA-24/40, OXA-48 and OXA-58 families that are mainly plasmid-encoded (Queenan and Bush 2007), and OXA-51 that is chromosomally encoded and intrinsic (naturally found) in *A. baumannii* (Manenzhe et al. 2015; Turton et al. 2006). Though, OXA-51 confers resistance or reduced susceptibility to carbapenems only when its expression is up-regulated by genetic reorganization (SMI P 8 2014). Class D enzymes are not inhibited by clavulanic acid or EDTA.

Whereas carbapenemase-producing bacteria are well characterized in high-income countries, little is known about them in Uganda and Africa at large (Manenzhe et al. 2015). Metallo-β-lactamase producing bacteria from a tertiary-care center in Nairobi, Kenya, was characterized in 2008, in a first report of VIM-2-producing *P. aeruginosa* in East Africa (Pitout et al. 2008). Furthermore, in a systematic review of 83 surveillance studies on carbapenemases in Africa (Manenzhe et al. 2015), it was revealed that most studies were from 15 of the 54 countries in Africa, mainly in Northern and Southern Africa with no report of negative results in studies that screened for carbapenemase-producing bacteria in humans in hospitals (Manenzhe et al. 2015). Indeed, prior to 2010 there were only seven reports of carbapenemase-producing bacteria in Africa with OXA-58, OXA-48, OXA-23, VIM-2 and VIM-4 documented as prevalent in carbapenemase-producing bacteria in outbreaks (Manenzhe et al. 2015). Carbapenemase-producing bacteria elaborating OXA-23, OXA-24, OXA-58, VIM-2 and IMP-1 were also isolated from hospital environments (Manenzhe et al. 2015). Recently in Uganda, we characterized carbapenem-resistant Enterobacteriaceae isolated from patients admitted at Mulago National Referral Hospital in Kampala (Okoche et al. 2015).

The aim of this study was to determine the prevalence of carbapenem resistant *P. aeruginosa* and *A. baumannii* at Mulago National Referral Hospital, and to establish whether the Mulago Hospital environment harbors carbapenem resistant Gram-negative rods. Herein we describe the susceptibility patterns and carbapenemase genes harbored by the isolates, as it might help in the global comparisons of resistance mechanisms of multidrug resistant Gram-negative bacteria.

## Methods

### Study setting and design

This study was conducted at Mulago National Referral Hospital in Kampala, Uganda. Mulago is a 1500-bed tertiary hospital belonging to the Ministry of Health, Uganda. With its free medical care, the hospital is highly attractive for the peri-urban low-income population around the capital Kampala where the infectious disease burden is high. The laboratory procedures were performed in Clinical Microbiology and Molecular Biology Laboratories of the Department of Medical Microbiology, College of Health Sciences, Makerere University.

Between February 2007 and September 2009, carbapenem resistant *P. aeruginosa* and *A. baumannii* were isolated from hospitalized patients at Mulago Hospital, in a laboratory surveillance study that aimed to identify carbapenem resistant Gram-negative rods at Mulago Hospital. Within this period, 869 clinical specimens from hospitalized patients were processed by the Clinical Microbiology Laboratory for culture and sensitivity testing (one specimen per patient). Specimens processed were blood (51), cerebral spinal fluid (49), tracheal aspirates (163), ear swabs (197), sputum (204), urine catheters (98), and pus (107). Following detection of *P. aeruginosa* and *Acinetobacter* species in the specimens, 80 samples were randomly collected from the hospital environment (surgical/medical wards including the intensive care units, ICUs); they included water (13), disinfectants like chlorhexidine gluconate (15), cleaning materials like mops and squeezers (15), sink swabs (22), and floor swabs (15). Water and disinfectants were sampled using sterile syringes with needles while swabs were used to sample sinks and wet floors.

### Identification of *P. aeruginosa* and *A. baumannii*

All clinical and environment samples were processed within 2 h of collection for identification of Gram-negative bacteria. Isolates were recovered on blood agar after incubating at 35–37 °C for 24–48 h. Then, single colonies were sub-cultured on MacConkey agar and incubated at 35–37 °C for 24–48 h. Isolates were presumptively identified based on colony morphology, Gram-staining properties and biochemical characteristics [oxidase, triple sugar iron (TSI), sulphur indole and motility (SIM), citrate, and urease tests]. Colonial morphological features (i.e. colonies with characteristic spreading pattern and serrated edges, fruity sweet-grape smell, and bright green color) were used to identify *Pseudomonas* isolates. Positive catalase and oxidase tests, negative TSI and glucose fermentation tests and growth at 42 °C were used to distinguish *P. aeruginosa* from other lactose non-fermenting Gram-negative rods. *Acinetobacter* was presumptively identified based on negative motility and catalase tests, negative oxidase and glucose fermentation tests, and inability to grow under anaerobic conditions.

### Identification of isolates to species level and drug susceptibility testing

To confirm *A. baumannii* to species level, PCR-amplification followed by DNA sequencing of the species-specific region of the *bla*_OXA-51_-like gene intrinsic to *A. baumannii* (Manenzhe et al. 2015; Turton et al. 2006) was performed, using chromosomal DNA extracted from presumptuously identified isolates as template. To confirm *P. aeruginosa* to species level, and to determine the antimicrobial susceptibility profiles of both *P. aeruginosa* and *A. baumannii*, minimum inhibitory concentrations (MICs) were performed using the ‘Phoenix Automated Microbiology System’ from Becton and Dickson, Franklin Lakes, NJ, USA. This system has combination testing panels that include (a) an identification (ID) side with dried substrates for bacterial identification; the ID portion of the Phoenix panels utilizes a series of conventional, chromogenic, and fluorogenic biochemical tests to identify the organism, (b) an antimicrobial susceptibility testing (AST) side with varying concentrations of antimicrobial agents, and (c) growth and fluorescent controls at appropriate well locations. Specimen processing and Gram staining procedure was performed according to the manufacturer’s guidelines.

Phoenix panels were inoculated with standardized inoculum according to the manufacturer’s guidelines; occasionally, minor modifications were performed as described elsewhere (Carroll et al. 2006). Briefly, after determining the Gram staining properties of the isolates, nonselective medium (blood agar) was used to prepare fresh pure cultures for isolate identification (ID) and antimicrobial susceptibility testing (AST). Isolates were inoculated into appropriate ID/AST combination panels for Gram-negative isolates that were loaded into the instrument and incubated at 35 °C. The ID broth was inoculated with bacterial colonies adjusted to a 0.5 McFarland standard, and the suspension poured into the ID side of the Phoenix panel after a 30 µl aliquot was removed and saved for AST. For AST, the Phoenix AST Indicator Solution was added to the AST broth tubes and mixed by inversion. The AST side of the combination panel contains 84 wells with dried antimicrobial panels and one growth control well. One free-falling drop of the AST indicator was added to the AST broth tube, and 30 µl of the standardized ID broth suspension transferred to the AST broth and incubated for 16 h at 35 °C. Samples were read automatically at the instrument’s set parameters.

Phoenix default MIC breakpoints were used; amikacin >32 µg/ml, gentamicin >8 µg/ml, imipenem >8 µg/ml, meropenem >8 µg/ml, ceftazidime >16 µg/ml, cefepime >16 µg/ml, aztreonam >16 µg/ml, piperacine-tazobactam 16/4 µg/ml, and ciprofloxacin >2 µg/ml for *P. aeruginosa*; amikacin >32 µg/ml, gentamicin >8 µg/ml, imipenem ≤1 µg/ml, meropenem ≤1 µg/ml, ceftazidime >4 µg/ml, cefepime >4 µg/ml, aztreonam >8 µg/ml, piperacine-tazobactam 4/4 µg/ml, and ciprofloxacin >2 µg/ml for *A. baumannii*. Quality control and maintenance were performed according to the manufacturer’s recommendations. Reference strains *Escherichia coli* ATCC 25922 and *P. aeruginosa* ATCC 27853 were included in the ID and AST Panels.

Following isolate identification and AST, carbapenem-susceptible isolates were retested with the disc diffusion susceptibility method (10 µg imipenem or 10 µg meropenem, BiolabZrt, Budapest, Hungary) to detect isolates with inhibition zone diameters of ≤25 mm as Clinical and Laboratory Standards Institute (CLSI) recommends screening them for carbapenemase production (Wikler 2006).

### Carbapenemase assays

To detect carbapenemase activity in carbapenem-resistant isolates, carbapenemase assays were performed with the modified Hodge test (MHT) and the imipenem-EDTA test using *K. pneumoniae* ATCC 700603 and *E. coli* ATCC 25922 as indicator strains (Okoche et al. 2015; Miriagou et al. 2010; Asthana et al. 2014). In the MHT assay, a 1:10 dilution of the indicator strains was made by diluting 0.5 ml of culture (at 0.5 McFarland) to 5 ml with sterile saline, which was streaked all over the Mueller–Hinton Agar (MHA) plate using a sterile swab. Then, 10 µg meropenem disk (BiolabZrt, Budapest, Hungary) was placed at the center of the MHA plate. Each test isolate was streaked in a straight line from the disk to the edge of the plate. *K. pneumoniae* ATCC BAA-1705 and *K. pneumoniae* ATCC BAA-1706 served as positive and negative controls, respectively. Strain BAA-1705 possesses a *K. pneumoniae* carbapenemase KPC-2 that is highly active against cephamycins, carbapenems, and to several extended spectrum beta-lactamases (ESBLs) (Broberg et al. 2013). Positive or negative results were interpreted according to the guidelines of CLSI and the UK Standards for Microbiology Investigations (SMI P 8 2014; Wikler 2006; Asthana et al. 2014).

To detect metallo-β-lactamase activity, the imipenem-EDTA double-disk synergy test was performed using an overnight liquid culture of the test isolate adjusted to a turbidity of 0.5 McFarland standard, and spread on the surface of MHA plates. Then, two discs with 10 µg imipenem each were placed on the agar 15 mm apart (center-to-center); 10 µl of 0.5 M EDTA was added to one of the imipenem disc to achieve a disc content of 1.5 mg. After incubating at 37 °C overnight, an increase in inhibition zone diameter of ≥5 mm in the EDTA-supplemented disc was interpreted as positive for metallo-β-lactamase production (SMI P 8 2014; Asthana et al. 2014).

Furthermore, carbapenem-susceptible isolates with disc inhibition zone diameters of ≤25 mm were also tested for carbapenemase activity (Wikler 2006); isolates with positive results were screened by PCR for carbapenemase genes.

### Detection of carbapenemase genes and integrons

As the patterns of carbepenemase-encoding genes differ significantly between countries (Woodford et al. 2010), PCR was performed to identify the profiles of these genes at Mulago Hospital. All carbapenem-resistant isolates were screened for metallo-β-lactamase genes (*bla*_IMP_, *bla*_VIM_, *bla*_SPM_, *bla*_NDM_) using PCR primers and conditions described elsewhere (Queenan and Bush 2007; Ma et al. 2015; Pitout et al. 2005; Fallah et al. 2013, 2014); *Acinetobacter* were screened for OXA-carbapenemase genes (*bla*_OXA-23_, *bla*_OXA-24_, *bla*_OXA-58_) using PCR primers and conditions described Ma et al. (2015). Class 1 integrons were detected with previously described primers 5′-CS (GGCATCCAAGCAGCAAG) and 3′-CS (AAGCAGACTTGACCTGA) that are specific to the 5′ and 3′ conserved segments (CS) of class 1 integrons (Levesque et al. 1995).

Chromosomal DNA used as templates in PCRs was extracted by the cetyltrimethyl ammonium bromide (CTAB) method (Andreou 2013; William and Feil 2012) and dissolved in 100 µl of sterile Tris-EDTA (TE) buffer. PCRs were performed in 10 µl volumes with 100 ng DNA template, custom Master-mix (1×), 0.5 µM each of forward and reverse primer, and 1.25 U Taq DNA polymerase, in a Techne TC-412 thermal cycler (Techne, UK). 5 µl of the amplified PCR product was analyzed by electrophoresis on 1 % agarose gels at 120 constant voltage for 1 h. PCR products were cleaned with the QIAquick PCR-purification kit (Qiagen, Hilden, Germany) and shipped to the United States for sequencing (ACGT Inc., Wheeling IL). Although our focus on PCR was screening mainly carbapenem-resistant isolates, carbapenem-susceptible isolates with disc inhibition zone diameters of ≤25 mm were also screened; for class 1 integron gene cassettes, all isolates were screened by PCR.

### Quality control

Negative controls for the PCR-amplified carbapenemase genes included reactions with only water (no DNA), and DNA template extracted from carbapenemase-negative strains *K. pneumoniae* DSMZ 9377, *E. coli* ATCC 25922 and *P. aeruginosa* ATCC 27853. Positive control reactions included template DNA extracted from carbapenemase-producing strains (*K. pneumoniae* Nr.8 for *bla*_NDM-1_, *K. pneumoniae* 714 for *bla*_OXA-48_) and a previously characterized *P. aeruginosa* clinical strain from Giessen for *bla*_IMP_ that was obtained from the Institute of Microbiology, Giessen, Germany [see Mushi et al. (2014)]. For *bla*_VIM_, the positive control strain was obtained from the RESET research collaboration, Germany [see Fischer et al. (2012)]. Additionally, targeted DNA sequencing of PCR-products and confirmation of sequenced amplicons through BLAST-searching at National Center for Biotechnology Information (NCBI) was performed. Isolates with sequenced amplicons that did not match sequences for the genes being studied were excluded.

## Results

### Patients and isolates

A total of 869 clinical specimens processed yielded 42 (5 %) *P. aeruginosa* isolates and 29 (3 %) *A. baumannii* isolates (one per specimen/patient). *P. aeruginosa* was isolated from tracheal aspirates (15 isolates), ear swabs (12 isolates), pus (7), sputum (5), blood (2) and urine catheter (1), while *A. baumannii* was isolated from ear swabs (8), tracheal aspirates (9), pus (4), sputum (2), blood (4), and cerebral spinal fluid (2), Additional file 1: Table S1. Mixed populations of *P. aeruginosa* and *Acinetobacter* species in the same sample were recovered from a total of nine specimens (1 %). The median age of participants with samples yielding *P. aeruginosa* was 18.5 years of whom 22 (52 %) were females while 20 (48 %) were males. On the other hand, the median age of participants with samples yielding *Acinetobacter* was 24 years of whom 13 (45 %) were females while 16 (55 %) were males. Furthermore, a total of 80 samples from the hospital environment that were processed yielded 46 (58 %) *P. aeruginosa* isolates and 11 (14 %) *A. baumannii* isolates. Overall, 88 *P. aeruginosa* and 40 *A. baumannii* were isolated (128 isolates in total), Table 1 and Additional file 1: Table S1.

**Table 1:** General characteristics of *P. aeruginosa* and *Acinetobacter* isolated from hospitalized patients and environment at Mulago Hospital in Kampala Uganda, 2007–2009

Species	Hospitalized patients (%)	Environment (%)	# isolates (%)
*P. aeruginosa*	42/869 (5 %)	46/80 (58 %)	88 (69 %)
*A. baumannii*	29/869 (3 %)	11/80 (14 %)	40 (31 %)
Total	71/869 (8 %)	57/80 (71 %)	128
Carbapenem resistance			
*P. aeruginosa*	10/42 (24 %)	15/46 (33 %)	25/88 (28 %)
*A. baumannii*	9/29 (31 %)	6/11 (55 %)	15/40 (38 %)
Subtotal	19/71 (27 %)	21/57 (37 %)	40/128 (31 %)
Multidrug resistance			
*P. aeruginosa*	34/42 (81 %)	25/46 (54 %)	59/88 (67 %)
*A. baumannii*	18/29 (62 %)	6/11 (55 %)	24/40 (60 %)
Subtotal	52/71 (73 %)	31/57 (54 %)	83/128 (65 %)
Metallo-β-carbapenemase genes			
*P. aeruginosa*	9/10 (90 %)	9/15 (60 %)	18/25 (72 %)
*A. baumannii*	1/9 (11 %)	1/6 (17 %)	2/15 (13 %)
Subtotal	10/19 (53 %)	10/21 (48 %)	20/40 (50 %)
OXA-carbapenemase genes			
*P. aeruginosa*	0	0	0
*A. baumannii* ^a^	7/9 (78 %)	5/6 (83 %)	12/15 (80 %)
Class 1 integrons^b^			
*P. aeruginosa*	13/42 (31 %)	20/46 (43 %)	33/88 (38 %)
*A. baumannii*	13/29 (45 %)	2/11 (18 %)	15/40 (38 %)
Subtotal	26/71 (37 %)	22/57 (39 %)	48/128 (38 %)

^a^Restricted to only carbapenem-resistant isolates; excludes *bla*_OXA51_ intrinsic to *A. baumannii*

^b^Gene cassettes found in class 1 integrons: aminoglycoside adenylyltransferase *ant(4′)*-*IIb* (3 isolates); trimethoprim-resistant dihydrofolate reductase *dfrA* (2 isolates); adenyltransferase *aadAB* (3 isolates); *QacE* delta1 multidrug exporter (2 isolates); quinolone resistance pentapeptide repeat protein *qnr* (1 isolate); and metallo-β-lactamase genes *bla*_VIM-4_-like, *bla*_IMP-19_-like, *bla*_IMP-26_-like (1 isolate each). Other genes detected were putative glucose dehydrogenase precursor and hypothetical genes common in *A. baumannii* class 1 integrons (3 isolates); non-ribosomal peptide synthetase (pyoverdine sidechain peptide synthetase) (1 isolate) in *P. aeruginosa* (see Additional file 1: Table S1)

### Carbapenem resistance

#### *P. aeruginosa*

Of the 42 *P. aeruginosa* isolates from patients, 10 were carbapenem resistant giving a prevalence of 1 % (10/869) in patients or 24 % (10/42) in isolates. Carbapenem resistant *P. aeruginosa* was isolated from pus (three patients with wounds), sputum (three patients with pneumonia), tracheal aspirates (three patients with hospital acquired pneumonia), and an ear swab (from a patient with otitis media), Additional file 1: Table S1. On the other hand, 15 (33 %, 15/46) *P. aeruginosa* from the environment were carbapenem resistant. Overall, 25 *P. aeruginosa* isolates were carbapenem-resistant, Table 1 and Additional file 1: Table S1. Furthermore, most isolates were multidrug resistant (resistance to three or more classes of antimicrobials); 73 % (52/71) and 54 % (31/57) for isolates from hospitalized patients and the environment, respectively (Table 1), with high resistance to ciprofloxacin (64 %, 27/42), ceftazidime (69 %, 29/42), gentamicin (69 %, 29/42), aztreonam (40 %, 17/42), and cefepime (55 %, 23/42) among isolates from hospitalized patients, Additional file 1: Table S1. However, compared to isolates from patients, the environment isolates were more susceptible to ciprofloxacin (43 %, 20/46), ceftazidime (41 %, 19/46) and gentamicin (37 %, 17/46), but more resistant to piperacillin-tazobactam (63 %, 29/46), Additional file 1: Table S1.

#### *A. baumannii*

Of the 29 *Acinetobacter* isolates from hospitalized patients, nine were carbapenem resistant giving a prevalence of 1 % (9/869) or 31 % (9/29) in isolates. Carbapenem resistant *Acinetobacter* was isolated from tracheal aspirates (four patients with hospital acquired pneumonia), ear swabs (three patients with otitis media), sputum (patient with pneumonia), and cerebral spinal fluid (patient with meningitis). For the environment, six isolates were carbapenem resistant (55 %, 6/11), Table 1 and Additional file 1: Table S1. Overall, 15 *A. baumannii* isolates were carbapenem resistant, Table 1 and Additional file 1: Table S1, and similar to *P. aeruginosa*, most *Acinetobacter* were also multidrug resistant; 62 % (18/29) in isolates from patients and 55 % (6/11) in isolates from environment, with high resistance to ciprofloxacin, gentamicin, piperacillin-tazobactam, ceftazidime and aztreonam, Table 1 and Additional file 1: Table S1.

#### Carbapenemase activity

Carbapenemase activity in carbapenem-resistant *P. aeruginosa* was 40 % (4/10) and 27 % (4/15) with MHT in isolates from patients and the environment, respectively; 60 % (6/10) and 67 % (10/15) with imipenem/EDTA test in isolates from patients and the environment, respectively. Carbapenemase activity in carbapenem-resistant *Acinetobacter* was 33 % (4/9) and 50 % (3/6) with MHT in isolates from patients and the environment, respectively; 22 % (2/9) and 17 % (1/6) with imipenem/EDTA test in isolates from patients and the environment, respectively. Hence, not all carbapenem resistant isolates studied were positive both with MHT and imipenem/EDTA test; however, the latter was more sensitive in detecting metallo-β-lactamase activity. Overall, 28 % (11/40) of carbapenem resistant isolates tested negative both with MHT and imipenem/EDTA test, Additional file 1: Table S1.

#### Carbapenemase genes

Metallo-β-lactamase genes detected in carbapenem-resistant *P. aeruginosa* were *bla*_IMP1_-like (36 %, 9/25), *bla*_IMP2_-like (4 %, 1/25), *bla*_VIM1_-like (32 %, 8/25), *bla*_SPM_-like (20 %, 5/25), and *bla*_NDM1_-like (4 %, 1/25), Table 2 and Additional file 1: Table S1. Most isolates with metallo-β-lactamase genes were positive with the imipenem-EDTA test, and *bla*_VIM-1_-like was the only metallo-β-lactamase that was detected in carbapenem-resistant *A. baumannii* at 13 % (2/15), Table 3 and Additional file 1: Table S1. As expected, OXA-carbapenemase genes were detected mainly in *A. baumannii*; *bla*_OXA-23_-like (60 %, 9/15), *bla*_OXA-24_-like (7 %, 1/15), and *bla*_OXA-58_-like (13 %, 2/15), Additional file 1: Table S1.

**Table 2:** Characteristics of carbapenem-resistant *Pseudomonas aeruginosa* isolates from hospitalized patients and environment at Mulago Hospital in Kampala, 2007–2009

Isolate no.	Month/year of isolation	Source	Isolation material	Resistance pattern	MHT	IMP-EDTA	Carbapenemase genes				Other genetic determinants
							*bla*_IMP_-like^a^	*bla*_VIM_-like^a^	*bla*_SPM_-like	*bla*_NDM_-like	Class 1 Integrons
J085	02/2008	Patient	Pus	CIP-CN-CAZ-ATM-AK-MEM	Yes	No	No	Yes	No	No	No
J105	03/2008	Patient	Pus	CN-CAZ-MEM	No	Yes	Yes	No	No	No	No
1665	07/2008	Patient	Ear swab	TZP-ATM-FEP-IMP	No	Yes	Yes	No	No	No	Yes
2545	06/2009	Patient	Sputum	CIP-CN-CAZ-ATM-AK-FEP-IMP	Yes	No	No	Yes	No	No	No
2608	06/2009	Patient	Tracheal aspirate	CIP-CN-CAZ-ATM-FEP-IMP-MEM	No	Yes	No	Yes	No	No	Yes
2665	08/2009	Patient	Sputum	CIP-ATM-AK-FEP-IMP-MEM	Yes	No	Yes	No	No	No	Yes
7545	09/2009	Patient	Pus	CIP-CN-TZP-CAZ-ATM-FEP-IMP-MEM	No	Yes	No	Yes	No	No	No
9608	09/2009	Patient	Sputum	CIP-CN-TZP-CAZ-ATM-FEP-IMP	Yes	Yes	Yes	No	No	No	Yes
PA0504	09/2009	Patient	Tracheal aspirate	CN-CAZ-AK-FEP-IMP-MEM	No	No	No	No	No	No	No
PA1688	09/2009	Patient	Tracheal aspirate	CIP-CN-TZP-CAZ-AK-FEP-IMP	No	Yes	No	Yes	No	No	No
J041-2	11/2009	Environment	Wet floor	CIP-CN-TZP-CAZ-ATM-AK-FEP-MEM	No	Yes	No	No	Yes	No	Yes
J049-1	11/2009	Environment	Wet floor	CIP-CN-TZP-CAZ-ATM-FEP-IMP-MEM	No	Yes	Yes	No	No	No	No
J052-1	11/2009	Environment	ICU mop	CIP-TZP-CAZ-ATM-FEP-IMP-MEM	No	Yes	No	No	Yes	No	Yes
J059	12/2009	Environment	ICU mop	CIP-CN-TZP-CAZ-ATM-AK-IMP-MEM	No	No	No	No	No	No	Yes
J081-1	12/2009	Environment	Squeezer	TZP-ATM-IMP-MEM	Yes	Yes	Yes	No	No	No	No
J081-3	12/2009	Environment	Water	CN-TZP-ATM-IMP-MEM	No	No	No	No	No	No	Yes
J096	12/2009	Environment	Sink	CN-TZP-CAZ-ATM-IMP-MEM	No	Yes	Yes	No	No	No	Yes
J851	12/2009	Environment	Sink	CIP-CN-TZP-CAZ-ATM-FEP-AK-IMP-MEM	No	Yes	No	No	Yes	No	Yes (*aadAB*)
J327	12/2009	Environment	Sink	CIP-CN-TZP-CAZ-ATM-FEP-AK-IMP-MEM	Yes	No	Yes	No	No	No	Yes
R017	02/2010	Environment	Sink	ATM-FEP-IMP-MEM	No	Yes	No	Yes	No	No	Yes
R007	02/2010	Environment	Wet floor	CIP-TZP-IMP-MEM	Yes	No	No	Yes	No	No	No
S6	02/2010	Environment	Wet floor	CIP-CN-TZP-CAZ-ATM-FEP-AK-IMP-MEM	No	No	No	No	No	Yes	No
S17	02/2010	Environment	Squeezer	ATM-AK-IMP-MEM	No	Yes	Yes	No	No	No	Yes
S10	02/2010	Environment	Wet floor	CIP-IMP	No	No	No	No	Yes	No	No
1942	02/2010	Environment	Sink	CIP-TZP-ATM-AK-FEP-IMP-MEM	Yes	No	No	Yes	No	No	Yes

Laboratory surveillance of 869 clinical specimens (blood, pus, tracheal aspirates, cerebral spinal fluid, sputum, ear swabs and urine catheter from hospitalized patients) for carbapenem resistant *Pseudomonas aeruginosa* and Acinetobacter species at Mulago National Referral Hospital in Kampala, 2008–2009. For comparison, 80 samples (water, disinfectants, sink-, mop, wet-floor- and squeezer swabs) from the hospital environment were studied

AK, amikacin; CN, gentamicin; IMP, imipenem; MEM, meropenem; CAZ, ceftazidime; FEP, cefepime; ATM, aztreonam; TZP, piperacillin/tazobactam; CIP, ciprofloxacin; Yes, positive; No, negative; MHT, modified Hodge test; IMP-EDTA, imipenem-EDTA double-disk synergy test

^a^Only *bla*_VIM1_-like and *bla*_IMP1_-like genes were detected

**Table 3:** Characteristics of carbapenem-resistant *Acinetobacter baumannii* isolates from hospitalized patients and environment at Mulago Hospital in Kampala, 2007–2009

Isolate no.	Month/year of isolation	Source	Isolation material	Resistance pattern	MHT	IMP-EDTA	Carbapenemase genes								Other genetic determinants
							*bla*_OXA-23_-like	*bla*_OXA-24_-like	*bla*_OXA-51_-like	*bla*_OXA-58_-like	*bla*_IMP_-like	*bla*_VIM_-like	*bla*_SPM_-like	*bla*_NDM_-like	Class 1 Integrons
J052-2	04/2008	Patient	Cerebral spinal fluid	CIP-CN-TZP-ATM-FEP-IMP-MEM	Yes	No	Yes	No	Yes	Yes	No	No	No	No	No
A081-7	04/2008	Patient	Ear swab	TZP-ATM-IMP	No	No	No	No	Yes	No	No	No	No	No	Yes
J093	08/2008	Patient	Ear swab	TZP-IMP-MEM	No	No	No	No	Yes	No	No	No	No	No	Yes
R100	07/2009	Patient	Sputum	CIP-CN-TZP-CAZ-ATM-IMP-MEM	No	Yes	No	No	Yes	Yes	No	Yes	No	No	No
1942	07/2009	Patient	Ear swab	CIP-IMP-MEM	Yes	No	Yes	No	Yes	No	No	No	No	No	Yes
C5	08/2009	Patient	Tracheal aspirate	CIP-TZP-IMP-MEM	No	Yes	Yes	No	Yes	No	No	No	No	No	Yes
S20	09/2009	Patient	Tracheal aspirate	CIP-CN-TZP-ATM-AK-IMP-MEM	Yes	No	No	No	Yes	Yes	No	No	No	No	Yes (*ant(4′)*-*Iib*)
2608-2	09/2009	Patient	Tracheal aspirate	CIP-CN-CAZ-ATM-FEP-IMP-MEM	No	No	Yes	No	Yes	No	No	No	No	No	No
AC1014	09/2009	Patient	Tracheal aspirate	CIP-CN-TZP-CAZ-ATM-IMP-MEM	No	No	Yes	No	Yes	No	No	No	No	No	Yes
J028-2	12/2009	Environment	Sink	CN-TZP-ATM-IMP-MEM	No	No	Yes	No	Yes	No	No	No	No	No	No
J044-2	12/2009	Environment	Sink	TZP-ATM-IMP	Yes	No	No	No	Yes	No	No	No	No	No	No
J046-2	12/2009	Environment	Wet floor	TZP-IMP	No	No	Yes	No	Yes	No	No	No	No	No	No
J054	12/2009	Environment	ICU mop	CIP-CN-TZP-CAZ-ATM-AK-IMP-MEM	No	Yes	No	Yes	Yes	No	No	Yes	No	No	No
J087-1	12/2009	Environment	ICU squeezer	MEM	Yes	No	Yes	No	Yes	No	No	No	No	No	Yes
J096-2	12/2009	Environment	Sink	CN-TZP-CAZ-ATM-IMP-MEM	Yes	No	Yes	No	Yes	No	No	No	No	No	No

Laboratory surveillance of 869 clinical specimens (blood, pus, tracheal aspirates, cerebral spinal fluid, sputum, ear swabs and urine catheter from hospitalized patients) for carbapenem resistant *Pseudomonas aeruginosa* and Acinetobacter species at Mulago National Referral Hospital in Kampala, 2008–2009. For comparison, 80 samples (water, disinfectants, sink-, mop, wet-floor- and squeezer swabs) from the hospital environment were studied

AK, amikacin; CN, gentamicin; IMP, imipenem; MEM, meropenem; CAZ, ceftazidime; FEP, cefepime; ATM, aztreonam; TZP, piperacillin/tazobactam; CIP, ciprofloxacin; SXT, trimethoprim/sulfamethoxazole; Yes, positive; No, negative; MHT, modified Hodge test; IMP-EDTA, imipenem-EDTA double-disk synergy test

Furthermore, two (8 %, 2/25) carbapenem resistant *P. aeruginosa* isolates lacked carbapenamase activity and they were also negative for the carbapenemase genes studied, Table 2 and Additional file 1: Table S1. Additionally, six (40 %, 6/15) carbapeneme-resistant *Acinetobacter* lacked carbapenemase activity, two of which (A081-7 and J093) were negative for carbapenemase genes (except *bla*_OXA51_-like), Table 3 and Additional file 1: Table S1. Two carbapenem-susceptible *P. aeruginosa* from the environment and seven carbapenem-susceptible *Acinetobacter* from patients carried carbapenemase genes, Additional file 1: Table S1. The carbapenem-susceptible *P. aeruginosa* isolates with genes were positive with the imipenem/EDTA test (implying metallo-β-lactamase activity) and had disc inhibition zone diameters of ≤25 mm for carbapenems.

#### Integrons

Class 1 integron amplicons ranging from 600 to 1500 bp were found in 38 % (48/128) of *P. aeruginosa* and *Acinetobacter*, of which 37 % (26/71) were found in clinical isolates while 39 % (22/57) in environment isolates. Further, gene cassettes were found only in 25 % (12/48) of the integron-positive isolates. These were aminoglycoside adenylyltransferase *ant(4′)*-*IIb* that encodes amikacin and tobramycin resistance (Sabtcheva et al. 2003) (3 isolates); trimethoprim-resistant dihydrofolate reductase *dfrA* that encodes resistance to trimethoprims (Huovinen 2001; Levings et al. 2006) (2 isolates); adenyltransferase *aadAB* (3 isolates); *QacE* delta1 multidrug exporter associated with multidrug resistance to antiseptics and disinfectants (Paulsen et al. 1993) (2 isolates); quinolone resistance pentapeptide repeat protein *qnr* (1 isolate); and metallo-β-lactamase genes *bla*_VIM-4_-like, *bla*_IMP-19_-like, and *bla*_IMP-26_-like that are associated with resistance to carbapenems (Kim et al. 2013; Libisch et al. 2004; Yamamoto et al. 2011) (1 isolate each). Gene cassettes were not found in 75 % (36/48) of the integron-positive isolates. Two isolates (PA1107 and S20) with the *ant(4′)*-*IIb* gene cassette were also resistant to amikacin while another isolate (AC1107) with a similar cassette exhibited intermediate resistance to amikacin, Additional file 1: Table S1. Except for isolates S20 (*A. baumannii*) and J851 (*P. aeruginosa*), all the detected gene cassettes occurred in carbapenem-susceptible isolates, Additional file 1: Table S1.

Other genes detected in integrons were the putative glucose dehydrogenase precursor and hypothetical genes common to *A. baumannii* class 1 integrons (3 isolates) and non-ribosomal peptide synthetase (pyoverdine sidechain peptide synthetase) (1 isolate) in *P. aeruginosa*, Additional file 1: Table S1.

## Discussion

In this study, we have described carbapenem-resistant *P. aeruginosa* and *A. baumannii* isolated from hospitalized patients and the environment at Mulago Hospital in Kampala, Uganda. As species identification is highly desirable to allow proper interpretation of the results (SMI P 8 2014), the isolates were successfully identified to species level using a rigorous methodology. While the isolates from hospitalized patients were from clinically relevant specimens referred to the diagnostic laboratory for culture and sensitivity testing, we could not rule-out colonization implying that some of the isolates might have been not clinically relevant, given the high rate of colonization on various body parts particularly by *Acinetobacter*. The purpose of this study was to detect carbapenem resistance and while important, confirmation of the isolates as infectious was beyond the scope of this study.

The prevalence of carbapenem resistant strains was relatively low among hospitalized patients; however, it was comparatively high in isolates particularly in the environment. This is of concern as the hospital environment is known to be a potential source of infection for hospitalized patients and health care workers. Furthermore, most isolates in this study were multidrug resistant with rates (i.e. 65 % overall; 73 % hospitalized patients, 54 % environment) comparable to those reported by Pitout et al. in Kenya (Pitout et al. 2008), Lee et al. in Korea (Lee et al. 2007) and Gu et al. in China (Gu et al. 2007), but lower than rates reported from Latin America (Labarca et al. 2016) and India (Uma Karthika et al. 2009). This could be due to differences in antibiotic usage between Uganda and the countries where these studies were done (Manikal et al. 2000).

It is important to note that carbapenemases are not the only mechanisms of acquired resistance to carbapenems; other resistance mechanisms in *P. aeruginosa* include upregulated efflux pumps and loss of the outer-membrane protein encoding gene *oprD* (SMI P 8 2014). As such, when screening for carbapenemases, two confounders have to be ruled-out (SMI P 8 2014); (a) not all carbapenem-resistant isolates produce a carbapenemase, (b) not all carbapenemase producers are resistant to carbapenems. In this study we did not screen for carbapenemases in carbapenem-susceptible isolates with disc inhibition zone diameters of >25 mm. Furthermore, while the automated systems efficiently detect carbapenem resistance, the inbuilt software in these systems is not always accurate at correctly inferring the presence of carbapenemases. The Phoenix BD expert system that we used is noted for its high sensitivity (ability to detect carbapenem resistance) but on the other hand, low specificity (ability to distinguish true carbapenemase producers) (SMI P 8 2014; Woodford et al. 2010). Hence, carbapenem-resistant isolates in this study were screened for carbapenemase production with the MHT and imipenem/EDTA test that have been effectively used to validate carbapenemase producers. These tests also can distinguish carbepenem-resistance mediated by carbapenemases from the one mediated by other mechanisms (SMI P 8 2014; Asthana et al. 2014). Our data showed that carbapeneme-resistance in this study particularly in *P. aeruginosa*, was mainly mediated by metallo-β-lactamases. Nevertheless, the reported low rates of carbapenemase activity (particularly with MHT) among carbapenem-resistant isolates could be due to the fact that the detection of carbapenemase activity in clinical isolates is challenging (Queenan and Bush 2007); the MHT assay suffers from low sensitivity, and interpretation of its results can be subjective (i.e. the identification of the clover-leaf indentation).

Molecular tests have been reliably used to confirm the presence of carbapenemase genes (SMI P 8 2014; Asthana et al. 2014). In this study, carbapenem resistance correlated well with carbapenemase gene detection; that is, 72 % (18/25) and 13 % (2/15) of carbapenem-resistant *P. aeruginosa* and *Acinetobacter* respectively, possessed metallo-β-lacatamase genes while all (15/15) carbapenem-resistant *Acinetobacter* carried OXA-carbapenemases. However, three carbapenem-resistant *Acinetobacter* isolates (A081-7, J093 and J044-2) carried only *bla*_OXA-51_–like gene intrinsic to *A. baumannii* and it likely does not confer carbapenem resistance unless it is up-regulated (SMI P 8 2014). Overall, organisms producing OXA-23, OXA-24, IMP, and VIM, the most prevalent carbapenemases in many settings (Manenzhe et al. 2015; Pitout et al. 2008; Amudhan et al. 2011), also appear to be the circulating *A. baumannii* and *P. aeruginosa* strains at Mulago Hospital. These enzymes also were the most prevalent among Enterobacteriaceae isolated from patients at Mulago (Okoche et al. 2015). Although only one isolate carried the *bla*_NDM-1_-like gene, its detection is of concern as NDM-1 producing bacteria are rapidly spreading worldwide (Manenzhe et al. 2015).

Furthermore, carbapenem resistant isolates (both *P. aeruginosa* and *A. baumannii* with carbapenemase genes), which lacked carbapenemase activity were also detected. As outlined above, this might reflect the difficulty in detecting carbapenemase production particularly in *Acinetobacter* (SMI P 8 2014); OXA-carbapenemases often have poor enzymatic activity leading to sub-optimal activity in some strains. Furthermore, carbapenem resistant isolates without carbapenemase genes and carbapenemase activity detected in this study alludes to the occurrence of additional non-carbapenemase resistance mechanisms in these isolates (outlined earlier) particularly in *P. aeruginosa* [e.g. upregulated efflux pumps, *oprD* loss (SMI P 8 2014)]. Non-carbapenemase mediated mechanisms need further study as we did not extensively characterize them in this study.

Integrons carry novel metallo-β-lactamase genes (Liakopoulos et al. 2013); in this study three isolates carried gene cassettes encoding VIM-4, IMP-19 and IMP-26 metallo-β-lactamases. Also, the other cassettes and genes identified in this study were previously confirmed to be associated with class 1 integrons (Levesque et al. 1995; Sabtcheva et al. 2003; Huovinen 2001; Levings et al. 2006).

## Conclusions

The prevalence of carbapenem resistant *P. aeruginosa* and *A. baumannii* is relatively low among hospitalized patients at Mulago Hospital. In the 2 year study period we detected only 40 carbapenem-resistant isolates combined. However, carbapenem-resistance prevalence is comparatively high in isolates and in the environment. The detection of carbapenem-resistant organisms in the environment is of concern, as the hospital environment is a potential source of infection for patients and health care workers. VIM-1, IMP-1, IMP-2, SPM and NDM-1 producing *P. aeruginosa* and OXA-23, OXA-58 and VIM-1 producing *A. baumannii* are the carbapenemase-producing strains circulating at Mulago Hospital.

## Additional file

10.1186/s40064-016-2986-7 Isolates, drug susceptibility profiles, carbapenemase genes, class 1 integrons and gene cassettes found.

## Abbreviations

*ant(4′)*-*IIb*: aminoglycoside adenylyltransferase gene
AST: antimicrobial Susceptibility Testing
ATCC: American type culture collection
BLAST: basic local alignment search tool
Carbapenemases (Queenan and Bush 2007): SME (for “*Serratia marcescens* enzyme”); IMI (for “imipenem-hydrolyzing β-lactamase”); NMC (for “not metalloenzyme carbapenemase”); GES (for “guiana extended spectrum”); KPC (for “*Klebsiella pneumoniae* carbapenemase”); VIM (for “verona integron-encoded metallo-β-lactamase”); IMP (for “active on imipenem”); SPM (for “Sao Paulo metallo-β-lactamase”); GIM (for “German imipenemase”); SIM (for “Seoul imipenemase”); and NDM (for “New Delhi metallo-β-lactamase”); OXA (for “oxacillin-hydrolyzing”)
CLSI: Clinical and Laboratory Standards Institute
CTAB: cetyltrimethyl ammonium bromide
*dfrA*: trimethoprim-resistant dihydrofolate reductase gene
EDTA: ethylenediammine tetra acetic acid
ESBLs: extended spectrum beta-lactamases
ID: identification
ICU: intensive care unit
MHA: Mueller–Hinton Agar
MHT: modified Hodge test
NCBI: National Center for Biotechnology Information
PCR: polymerase chain reaction
SIM: sulphur indole and motility
TE: Tris–EDTA buffer
TSI: triple sugar iron
